# Endometriosis is not the endometrium: Reviewing the over-representation of eutopic endometrium in endometriosis research

**DOI:** 10.7554/eLife.103825

**Published:** 2025-05-20

**Authors:** Kate Gunther, Teagan Fisher, Dongli Liu, Jason Abbott, Caroline Elizabeth Ford

**Affiliations:** 1 Gynaecological Cancer Research Group, Lowy Cancer Research Centre, School of Clinical Medicine, Faculty of Medicine & Health, UNSW Sydney Sydney Australia; 2 Gynaecological Research and Clinical Evaluation (GRACE) Unit, Royal Hospital for Women Sydney Australia; 3 National Endometriosis Clinical and Scientific Trials (NECST) Network Sydney Australia; 4 Discipline of Women’s Health, School of Clinical Medicine, UNSW Sydney Sydney Australia; 5 Translational Oncology Group, School of Life Sciences, Faculty of Science, University of Technology Sydney Sydney Australia; University of Queensland Australia; University of Michigan-Ann Arbor United States

**Keywords:** endometriosis, endometrium, biospecimen, data repositories

## Abstract

As a heterogenous disease with likely multiple pathogeneses and as-yet-undefined subtypes, progress in endometriosis treatment is currently limited by a lack of appropriate models and cohorts for research. Almost half of all publicly available datasets labelled as ‘endometriosis’ do not represent true disease as they are based on eutopic endometrium. Eutopic endometrial cells and tissues are frequently being used to represent endometriotic lesions, despite the unequivocal differences at both the tissue and cellular levels. As preclinical endometriosis research increases, it is important that the unique cellular and molecular profiles of endometrium and endometriosis are distinguished. Whilst each of these biospecimens can provide invaluable information to better understand disease aetiology and identify targets for diagnosis and treatment, it is imperative that the appropriate biospecimen and model are used to answer the relevant research question because endometriosis is not the endometrium.

## Introduction

The perception that endometriosis tissue represents ‘ectopic endometrium’ is a fundamentally flawed and outdated concept, and an overall misnomer. Nevertheless, the clinical definition of endometriosis currently remains hinged upon endometrium, with histopathological diagnostic criteria dependent on the identification of endometrium-like glandular epithelium admixed with stromal cells. Despite being a heterogenous disease with multiple proposed pathogeneses (Signorile et al., 2022) and as-yet-undefined subtypes, the majority of preclinical research in endometriosis remains focused on an endometrium-dependent aetiology. The effect of this narrowmindedness, particularly in experimental design, is already evident in the literature, resulting in unreliable reproducibility, an overall stagnation of knowledge, and wasted resources.

Endometriosis is under-represented in medical research outputs despite its high prevalence. Conservative estimates from the World Health Organization put endometriosis as 3.4 times more common than dementia (World Health Organization, 2023a; World Health Organization, 2023b). However, while there are 37,384 research articles on endometriosis in total to date, dementia had 34,546 articles published in 2023 and 2024 alone (https://www.webofscience.com term = endometriosis; term = dementia, accessed February 5, 2025). At the World Economic Forum in January 2025, endometriosis was listed as one of nine conditions in women leading to the greatest personal, societal, and economic burden globally (World Economic Forum, 2025). Lack of funding remains one of the most substantial barriers to obstetrics and gynaecology research, which has consistently been one of the lowest funded NIH specialties in the last decade (Schlafly and Sebro, 2022). This may be on the precipice of change, with the NIH budget for endometriosis research projected to double between 2020 and 2024 (National Institutes of Health, 2024), during which time there has been a marked increase in both overall and interdisciplinary clinical trials (Xu et al., 2024).

It is imperative that this funding is funnelled into the generation of biologically relevant research. The scant clinical translation of endometriosis research has previously been reviewed in the context of the lack of physiologically relevant in vivo models (Malvezzi et al., 2020), and the prevalence of invalidated in vitro models (Romano et al., 2020). This review aims to critically appraise the rationale of current preclinical research, particularly in the selection of models and controls for experimental research and their reliance on eutopic endometrium. The literature will be reviewed to summarise key biological distinctions between endometrium and endometriosis, and examples will be given detailing how their similarities and differences could be utilised to answer three key research priorities concerning diagnosis and prognosis, disease aetiology, and treatment.

### Eutopic endometrium is over-represented in endometriosis research

Endometriosis is defined as a chronic inflammatory disease marked by the presence of lesions in extrauterine locations which microscopically represent endometrium-like epithelium and/or stroma (Tomassetti et al., 2021). Endometriosis lesions are cellularly heterogeneous, with significant contribution from endothelial, myeloid, and lymphocyte populations (Tan et al., 2022). The most common classification system defines phenotypes according to their macroscopic appearance during surgery, defined as superficial (<5 mm invasion of the peritoneum or serosa), deep infiltrating (>5 mm invasion), or ovarian cystic endometriomas. While peritoneal and endometrioma phenotypes can co-exist, the phenotype of peritoneal lesions is classified according to the ‘most severe’ lesion/s observed; and any extrapelvic disease is considered deep infiltrating by definition. Whether endometriomas and peritoneal lesions should be considered distinct entities remains contentious. While single-cell transcriptomic analysis of endometriotic lesions has revealed that endometriomas are highly enriched for stromal cells compared to peritoneal lesions (Fonseca et al., 2023), the sample sizes remain small, and cellular proportions are heterogenous between patients, even within the same phenotype (Tan et al., 2022). Furthermore, investigations of intrapatient mutational signatures have demonstrated that both peritoneal lesions and endometriomas can share common somatic mutations, providing evidence for potential clonal development of disease from a common initiation in some patients (Praetorius et al., 2022). Attempts to molecularly subtype endometriosis revealed distinct transcriptional signatures relating to fibrosis or immune dysfunction, which are independent of surgical phenotype (Wang et al., 2023). It is possible that molecular and cell proportion differences observed between endometriosis phenotypes, namely between peritoneal endometriosis versus endometrioma, arise from innate variations, or are emphasised over time due to unique microenvironmental pressures. It is likely that ongoing efforts to improve classification beyond macroscopic appearance, including molecular classification, will reveal further subtypes of endometriosis.

By contrast, eutopic endometrium, defined as endometrium present in the native uterine cavity in those with endometriosis, is a dynamic and complex multicellular structure, with key hormonal and immunological functions beyond the reproductive role of the functional layer (Garcia-Alonso et al., 2021). Despite the structural similarities between eutopic endometrium and endometriosis lesions, there have been calls to shift the collective focus of endometriosis research away from comparing the two tissues (Colgrave et al., 2021).

### Critical analysis of available datasets representing endometriosis

**Figure 1.**
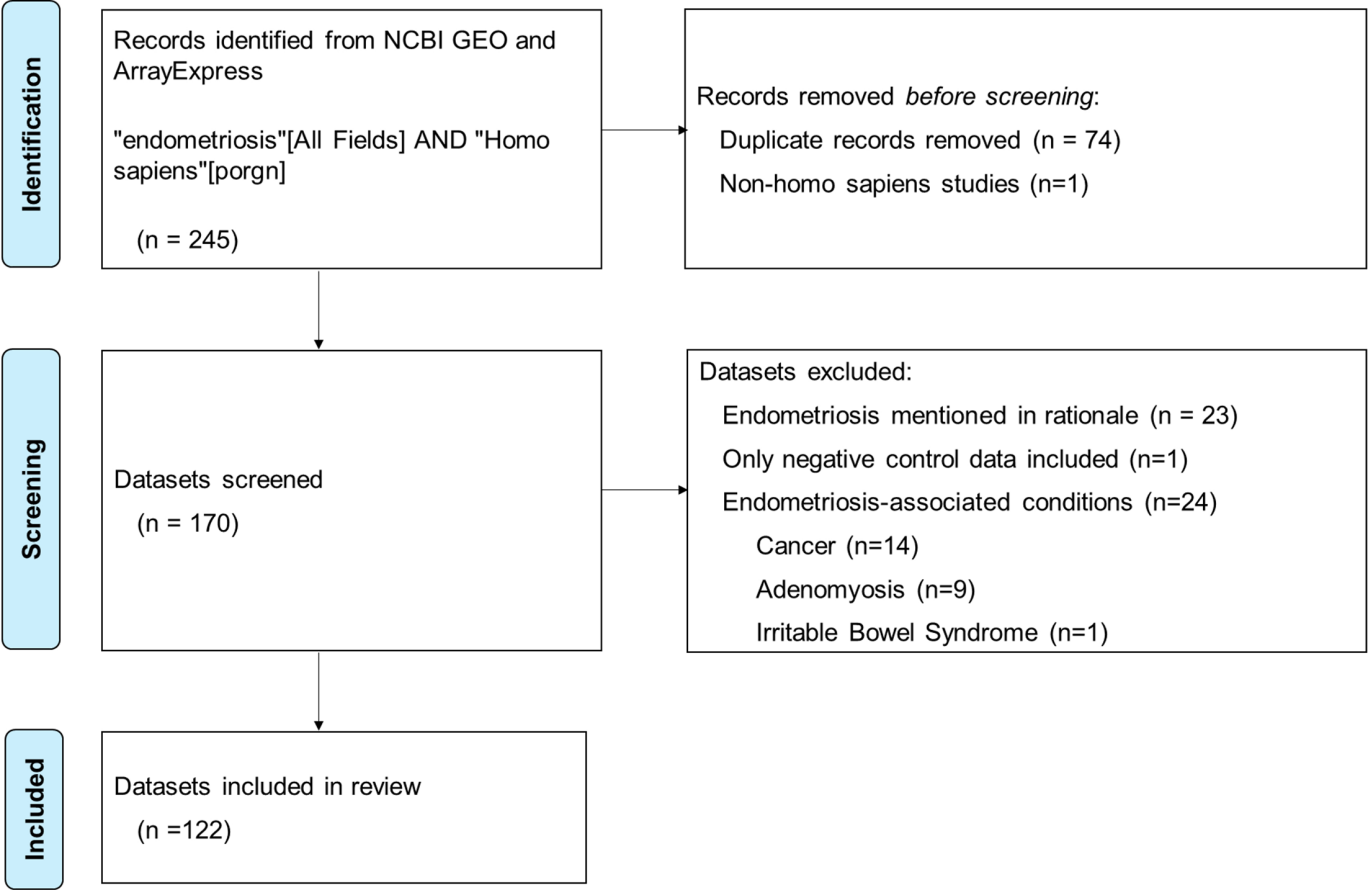
Flow chart of search and screening process for review of publicly available endometriosis datasets.

The ‘Big Data Revolution’ and the advent of genomics have prompted a substantial rise in secondary data analysis, particularly the reuse of omics data from gene expression profiling databases like the Gene Expression Omnibus (GEO) and other data-sharing sites such as ArrayExpress. This is particularly evident in endometriosis research, where strict time and budgetary restraints mean that many researchers rely on secondary data for some or all of their research design. To investigate the role of methodological bias in endometriosis research, a review of publicly available endometriosis data sourced from NCBI GEO and ArrayExpress as of January 27, 2025, was conducted, yielding 245 results (Figure 1). After screening, 122 datasets were reviewed for biospecimen source.

**Figure 2.**
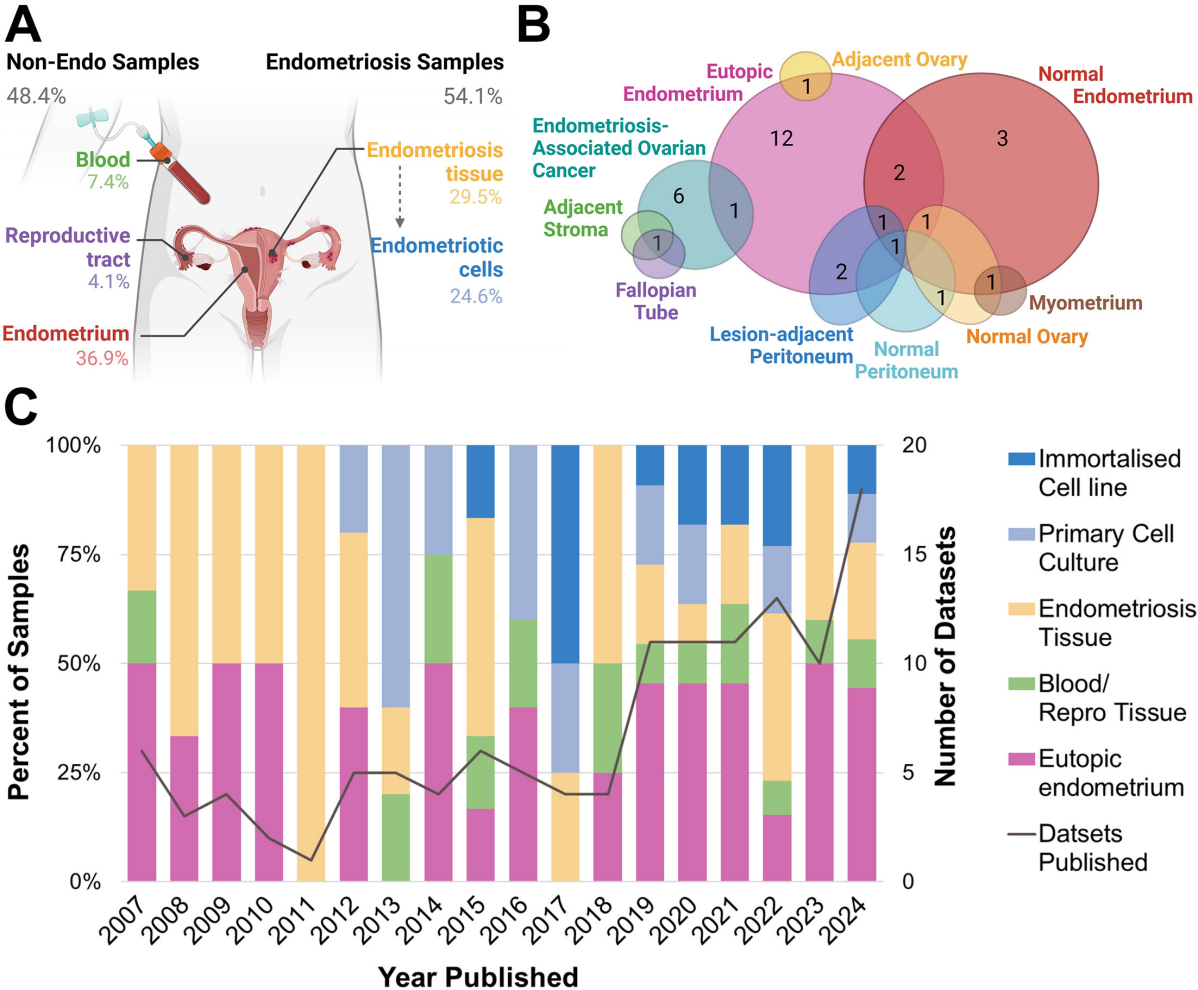
Biospecimen sample source for publicly available endometriosis datasets. (**A**) Overall percentage of each sample type according to biospecimen source, whether from endometriosis or non-endometriosis (non-endo). (**B**) Type of biological control for endometriosis tissue datasets. (**C**) Breakdown of biospecimen source and number of published datasets per year, 2007–2024. All primary cell cultures and immortalised cell lines derived from endometriosis tissues (endometriotic cells). Note percentages do not amount to 100.00 in (A) due to three datasets containing more than one biospecimen type. This figure was created using BioRender.com.

Most notably, 45/122 (36.89%) datasets contained eutopic endometrium only (Figure 2A), more than any other biospecimen type. This includes endometrial tissue from both curettage and menstrual effluent (25/45, 55.56%), and endometrial cells, including stromal, epithelial, organoids derived from menstrual effluent and bacterial cells (20/45, 44.44%). There were 14 datasets which contained non-endometriosis or non-endometrium from patients, including circulating blood or blood vessel tissue (9/122, 7.38%) and fluid, cells and tissues from the reproductive tract, including follicular fluid, tubal fluid and tissue, choriodecidua, and granulosa cells (5/122, 4.10%). When combined with the endometrium samples, almost half of all biospecimens listed as ‘endometriosis’ had no representation of true endometriotic disease (59/122, 48.37%). When considering the year of publication, there has been a steady increase in the overall number of datasets published each year (Figure 2C). Despite this, annual representations of endometrium-only datasets are persistent at approximately 50%. There has been an increase in the number of endometriotic cell samples since 2012, which have shown a trend towards immortalised cell lines since 2017.

For datasets containing endometriotic cells, one dataset contained endometriosis organoids of epithelial cell origin (1/17, 6.25%), while all primary cells were stromal (16/16, 100%). By contrast, all immortalised cell lines were epithelial (13/13, 100%). The bias towards primary stromal endometriotic cells is likely contributed to by the enrichment of stromal cells in endometriotic tissues (Tan et al., 2022; Fonseca et al., 2023), as well as the difficulty in growing epithelial cells in vitro. Importantly, this demonstrates the lack of diversity in available immortalised cell lines to model endometriosis. Endometriosis phenotype was not recorded in a substantial portion of tissue (4/36, 11.43%) and primary cell culture datasets (5/17, 29.41%). For datasets where phenotype was recorded, endometriomas were disproportionately represented, constituting 70.59% of primary cell samples and 72.22% of tissue datasets (endometrioma, multiple phenotypes containing endometrioma, Table 1), despite an overall prevalence of approximately 30% amongst endometriosis lesions (Bourdon et al., 2024). This over-representation may be a result of larger lesion volume and therefore increased output, or a shared interest from those investigating endometriosis-associated ovarian cancers.

**Table 1.** Review of publicly available endometriosis datasets.

	Number	Percentage	Datasets
**Endometrium only**	**45**	**36.89**	**Endometrial datasets**
Tissue	25	55.56	GSE101176, GSE11768, GSE107469, GSE120103, GSE130028, GSE130435, GSE134052, GSE134056, GSE135485, GSE135640, GSE139954, GSE145702, GSE14808, GSE153739, GSE153740, GSE172172, GSE174305, GSE167946, GSE17504, GSE188915, GSE19834, GSE216255, GSE202571, GSE203191, GSE223817, GSE232713, GSE31683, GSE35287, GSE40007, GSE51981, GSE6364, GSE7305, GSE73622, GSE73950, GSE94414, GSE85701, GSE7846, GSE262037, GSE193928, GSE268541, GSE212787, GSE272606, GSE275002, E-MTAB-14039, E-MTAB-14058
Cells	20	44.44	
**Immortal cell line**	**13**	**10.66**	**Cell line datasets**
12Z	7	53.85	GSE114332, GSE68104, GSE86572, GSE97373, GSE261931, GSE184431, GSE152661, GSE157735, GSE174741, GSE190549, GSE202661, GSE210201, GSE279835
EEC16	5	38.46	
iHEECs	1	7.69	
**Non-lesion endometriosis**	**14**	**11.48**	**Non-lesion endometriosis**
Blood and blood vessels	9	64.29	GSE168214, GSE57832, GSE205494, GSE220787, GSE192636, E-MEXP-1251, GSE77182, GSE153813, GSE133867, GSE182983, GSE69310, GSE46735, GSE279435, GSE124010
Reproductive tract	5	35.71	
**Primary tissue culture**	**17**	**13.93**	**Primary culture datasets**
Primary cells	16	94.12	GSE124010, GSE121406, GSE135122, GSE136412, GSE31515, GSE40186, GSE44207, GSE47361, GSE58178, GSE67524, GSE75427, GSE87810, GSE243158, GSE132464, GSE269530, GSE168902, GSE118928
Primary organoids	1	6.25	
Endometrioma	12	70.59	
No phenotype listed	5	29.41	
Stromal	16	94.12	
Epithelial	1	5.88	
Without biological control	3	17.65	
With biological control	14	82.35	
Normal endometrium only	6	42.86	
Eutopic endometrium only	5	35.71	
Both eutopic and normal	3	21.43	
Additional controls	0	0.00	
**Endometriosis tissue**	**36**	**29.51**	**Endometriosis tissue datasets**
Endometrioma only	19	52.78	GSE279835, GSE11691, GSE197928, GSE12768, GSE141549, GSE15309, GSE157153, GSE16079, GSE179640, GSE185273, GSE196748, GSE201912, GSE213216, GSE226575, GSE230956, GSE23339, GSE239685, GSE246202, GSE25628, GSE26346, GSE37837, GSE5108, GSE56414, GSE57545, GSE71477, GSE7307, GSE86534, GSE99949, GSE229735, GSE105764, GSE248593, GSE281569, GSE247695, E-MTAB-694, GSE94414
Superficial only	1	2.78	
Deep infiltrating only	1	2.78	
Peritoneal, unknown depth	3	8.33	
No phenotype listed	4	11.11	
Multiple phenotypes	8	22.22	
Without biological control	0	0.00	
With biological control	36	100.00	
Normal endometrium only	3	8.33	
Eutopic endometrium only	13	36.11	
Endometrium unknown source	2	5.56	
Both eutopic and normal	2	5.56	
Compared to EAOC	6	16.67	
Additional controls	10	27.78	

EAOC, endometriosis-associated ovarian cancer.

Full summary of the reviewed publicly available datasets, their biospecimens, and any associated publications can be found in Supplementary file 1.

All endometriosis tissue datasets contained a biological control, while only 82% of primary cell experiments did (Table 1). Of these controls, the majority of endometriosis tissue biospecimens were compared to eutopic endometrium as a biological control (13/36, 36.11%), while for three datasets, endometriosis lesions were compared to ‘healthy’ (non-endometriosis) endometrium only (3/36, 8.33%). When considering both the datasets containing only endometrium-derived samples and the presence of eutopic endometrium as a biological control, the presence of endometrium far exceeds that of endometriosis (90/122, 73.77% vs 66/122, 54.10%). The use of non-endometrium tissue as a biological control was varied (Figure 2B). This includes the use of normal tissues such as peritoneum, ovary, fallopian tube and myometrium, as well as tissue directly adjacent to endometriosis lesions such as stromal tissue, ovary, or peritoneum. Collectively, these adjacent tissues, representing the only microenvironment-relevant controls in the entire cohort, accounted for less than 5% of datasets analysed (6/122, 4.92%).

The lack of diverse and phenotypically defined models of endometriosis has been described previously (Gołąbek-Grenda and Olejnik, 2022) and is the rationale behind the development of defined endometriosis phenome and biospecimen collection procedures by the World Endometriosis Research Foundation (Fassbender et al., 2014; Rahmioglu et al., 2014). However, the over-representation of eutopic endometrium as an experimental model of endometriosis has been largely unexplored until now. The effect of this data bias is already apparent in the literature. Secondary use of transcriptional datasets derived from eutopic endometrium has been used to identify potential therapeutic targets for endometriosis, despite samples not representing endometriotic disease (Nayak et al., 2022). Attempts to molecularly characterise endometriosis and immune infiltration of the disease have also used datasets composed entirely of eutopic endometrial tissue (Lv et al., 2023). While there are similarities between eutopic and ectopic tissues, it is inadvisable to use one as a surrogate for the other.

### Eutopic endometrium should not be used to represent endometriosis lesions

The frequency with which eutopic endometrium is used to represent endometriosis lesions by proxy is likely due to the ease of access to endometrial tissue via menstrual effluent or curettage versus surgically retrieved endometriotic samples. However, there are profound differences between endometrial and endometriotic tissues.

Deconvolution of cell populations has shown distinct cellular compositions of the two tissues, with epithelial cell populations significantly reduced in ectopic tissues when compared to eutopic, and the inverse true of stromal cells (Tan et al., 2022; Fonseca et al., 2023). Endometriotic cells demonstrate increased adherence (Delbandi et al., 2013), migration (Dai et al., 2019), and invasion (Kao et al., 2011) capacity compared to endometrial cells, owing to increased epithelial-to-mesenchymal-transition (Zubrzycka et al., 2023) and decreased matrix metalloproteinase expression (Maoga et al., 2023). Transcriptional analysis has shown a coordinated pathway change in endometriotic tissue in favour of inflammatory activation, collagen formation, and extracellular matrix degradation (Fonseca et al., 2023). Ectopic endometriotic cells are also more proliferative both in vivo (Li et al., 1993) and in vitro (Wang et al., 2021), demonstrating a pro-survival phenotype of increased S-phase and decreased G0 cell cycle phase (Rashidi et al., 2023). Upon cell death, endometriosis cells are more likely to undergo inflammatory modes including pyroptosis (Xu et al., 2023) and ferroptosis (Li et al., 2021b; Zhang et al., 2022), instead of Bcl-2/Bcl-xL/Caspase-3 mediated apoptosis (Delbandi et al., 2020) or autophagy (Huang et al., 2021) as is observed in eutopic endometrium. At a tissue level, endometriotic lesions have higher levels of reactive oxygen species and hypoxia compared to matched endometrium (Dai et al., 2019; Yerlikaya et al., 2016), mediating both angiogenesis (Fu et al., 2018) and a metabolic reprogramming analogous to the Warburg effect (Kasvandik et al., 2016; Chen et al., 2023).

There is significantly increased immune cell infiltration in ectopic endometriosis tissues (Tan et al., 2022) and acute dysregulation of both innate and adaptive immune function. Innate myeloid populations, including monocytes, inflammatory (formerly M1), and regulatory (formerly M2) macrophages and resting mast cells, are all elevated in lesions compared with paired eutopic endometrium, while fewer activated dendritic cells can be found (Zhou et al., 2023). Functionally, endometriosis-associated immune cells display altered signalling (Tan et al., 2022) and antigen-presenting ability (Zhu et al., 2023), which could explain the reduced immunosurveillance and phagocytic activity of these cells in vitro (Lukács et al., 2021). Similarly, there is an increase in resident adaptive lymphocytes such as memory B cells, plasma cells, and both resting and activated CD4+ and CD8+ cells, with a reduction in the number of regulatory T-cells and both resting and activated natural killer cells (Zhou et al., 2023; Zhu et al., 2023) in endometriosis compared to matched eutopic endometrium. This orchestrated immune dysregulation extends beyond the immediate microenvironment, creating a systemic proinflammatory milieu in favour of a Th2 cytokine response (Xia et al., 2023; Olkowska-Truchanowicz et al., 2021).

Despite the marked differences between eutopic endometrium and ectopic endometriosis tissues, this does not negate their utility in preclinical endometriosis research. To illustrate the role of diverse biospecimen collection in endometriosis research, three major focuses of the literature were reviewed in the context of how both eutopic and lesion-adjacent microenvironmental specimens could be utilised. There remains no simple diagnostic test for endometriosis, and while efforts are being made to improve access and accuracy of imaging, laparoscopy remains the mainstay of endometriosis diagnosis. Treatment options remain limited to surgical, hormonal, or analgesic management, and there is a significant unmet clinical need for novel therapies. While there are multiple proposed theories of pathogenesis, a clear cause of endometriosis remains elusive. These topics – diagnosis and prognosis, disease aetiology, and treatment – have also been identified as the top research priorities by endometriosis patients (Armour et al., 2023).

### The value of researching eutopic endometrium

Opening sentences of publications on endometriosis frequently contain the same pervasive definition: “a chronic inflammatory condition wherein cells *similar to* the endometrium are found outside the uterus”. Such is the similarity that the term endometriosis was selected due to the morphological likeness (Sampson, 1925). The eutopic endometrium has been extensively profiled in endometriosis research, and whilst it remains critical that the pathological distinction between endometrium and endometriosis is identified, there are clear benefits in understanding the eutopic endometrium to provide a comprehensive picture of endometriosis in its entirety.

### Diagnosis and prognosis

Eutopic endometrium from people with endometriosis demonstrates profound structural, transcriptomic, methylomic, proteomic, and immunologic differences from ‘healthy’ (control) endometrium (Tan et al., 2022; Adamczyk et al., 2022; Wu et al., 2021; Méar et al., 2022; Prašnikar et al., 2020). Understanding these differences was of significant interest in the development of an endometrial biopsy-based diagnostic test, of which neural fibre marker PGP 9.5 demonstrated comparable accuracy to traditional surgical diagnosis (Gupta et al., 2016). Since this time, greater interest in non-invasive endometrial sampling has arisen.

Endometrial fluid can be aspirated from the uterine cavity transcervically in an outpatient setting, thereby increasing the accessibility to, and greatly reduce the cost of, diagnosis. Collecting endometrial fluid directly from the uterine cavity has shown utility in diagnosing endometrioma by lipidomic profiling (Domínguez et al., 2017). Endometrial lavage has also proven to be a viable biospecimen for cytokine and exosome studies. Cytokine profiles in endometrial or cervicovaginal lavage samples, namely IL-1A, IL-6, and Regulated on Activation, Normal T cell Expressed and Secreted (RANTES), have shown predictive capacity to identify endometriosis of variable phenotypes versus symptomatic controls (Llarena et al., 2020; Jimenez et al., 2024). Within endometriosis cases, distinct inflammatory signatures were observed between early (I/II) versus late (III/IV) stage, and endometrioma versus peritoneal lesions (Jimenez et al., 2024). Exosomes from endometrial lavage have identified a specific microRNA (miRNA), miR-210-3p, as upregulated in those with endometrioma, irrespective of menstrual phase (Jiang et al., 2022). These miRNAs have also been successfully isolated from extracellular vesicles collected from both cervical brush samples and vaginal swabs in endometriosis patients (Paterson et al., 2024).

Similar attempts to establish diagnostic signatures have emerged by utilising menstrual effluent (Shih et al., 2022; Nayyar et al., 2020; Miller et al., 2022; Warren et al., 2018). A clinical trial is currently underway to develop a screening algorithm based on the immune cell population and transcriptional profile of eutopic endometrium for people with endometriosis (NCT05601596). Proteomic profiles of normal endometrium and eutopic endometrium are also being investigated for diagnostic efficacy (NCT06214260). The use of menstrual blood for disease diagnosis or management is not new. Menstrual blood is already used for clinical monitoring of glucose control in diabetes via FDA-approved menstrual pad, Q-Pad (Naseri and Therkelsen, 2021; Naseri and Therkelsen, 2017). There is great promise for similar models to use menstrual effluent as a non-invasive biospecimen in the context of endometriosis (Tindal et al., 2024).

### Disease aetiology

Comparison of eutopic and ectopic tissue somatic mutation burden supports an endometrial origin of endometriosis lesions, though the prevalence and clinical significance of this aetiology remain unresolved. An endometrial origin of endometriosis has been observed across all major phenotypes (Noë et al., 2018; Li et al., 2021a). Individual case studies show that disease evolution is unique, with heterogenous cell phylogenies between patients (Li et al., 2021a). Further research comparing acquired somatic mutations between endometrium and endometriosis may reveal the diversity of clonal lesion development and their recurrence, as well as inform how many people with endometriosis have endometrium-dependent pathogenesis.

Both epithelial (Suda et al., 2018; Li et al., 2014) and mesenchymal (Suda et al., 2019; Noë et al., 2018; Li et al., 2021a) endometriosis cells have been traced to an endometrial origin. It is likely that these endometriosis-initiating cells represent endometrial stem cells, with markers of both endometrial epithelial (Tan et al., 2022; Fonseca et al., 2023; Garcia-Alonso et al., 2021; Valentijn et al., 2013; Zeitvogel et al., 2001) and mesenchymal (Tan et al., 2022) stem cells identified in endometriosis tissue. When isolated from lesions, endometriosis stem cells demonstrate the same colony-forming efficiencies and cellular reprogramming capacities as eutopic endometrial stem cells, able to differentiate into adipocytes, osteoblasts, chondrocytes, cardiomyocytes, and neural cells (Kao et al., 2011; Li et al., 2023).

It is theorised that these endometrium-derived endometriosis-initiating stem cells are transported to ectopic sites via retrograde menstruation (Masuda et al., 2021), iatrogenesis (Benedetto et al., 2022; Neamtu et al., 2022), lymphatic (Velho et al., 2023; Jerman et al., 2020), or haematological spread (Wang et al., 2024; Kiss et al., 2020; Pospisilova et al., 2019), with case studies supporting each theory. Upon transport to ectopic locations, it is postulated that decreased apoptosis (Prašnikar et al., 2020; Schwalie et al., 2024), increased proliferation (Tan et al., 2022), and inflammation (Tan et al., 2022; Shih et al., 2022) observed in eutopic endometrium give these disease-initiating cells a survival advantage and facilitate their adhesion and invasion of parenchymal tissues such as peritoneum or ovarian epithelium.

Understanding which aetiology, or aetiologies, has led to disease initiation could have implications in disease management, including prevention. Localised progestin treatment via levonorgestrel intrauterine systems (LNG-IUS) has been shown to decrease symptom recurrence (Kim et al., 2022) and reoperation rates in endometrioma (Choi et al., 2023). While LNG-IUS use is associated with reduced menstrual bleeding or complete amenorrhea (Parks et al., 2020), this action is independent of any effect on circulating estradiol (Tasci et al., 2009) and minimal effects on ovulation (Apter et al., 2014). This may support the notion that recurrence of both pain and lesions in some patients is initiated by de novo disease from endometrium-derived cells (Vercellini et al., 2024b; Yela et al., 2021; Tsuboshima et al., 2023). Whether this same rationale could be applied to primary prevention of endometriosis remains to be seen. The current discrepancy between age of symptom onset and achieving diagnosis has resulted in a poor understanding of early lesion development. Whether early intervention targeting endometrium, particularly during adolescence, could prevent or reduce disease burden remains theoretical (Vercellini et al., 2024a).

### Treatment

The specificity of any treatment relies on the identification of therapeutically actionable differentially expressed biomarkers in the tissue of interest. In the context of endometriosis, eutopic endometrium is arguably the most valid choice as a reference tissue due in large part to the transcriptional similarity of the two tissues. A meta-analysis of RNA expression differences between patient-matched endometriosis and eutopic endometrium found an overall difference of only 4.74% (15,234/321,149 genes) (Riaz et al., 2024). The 11 studies amounted to 116 participants, one of which did not report disease phenotype, and the remaining disproportionately represented endometriomas (71/111, 63.96%).

These similarities affect the development of therapeutics for endometriosis. For example, the overall goal of hormonal agents is to induce a hypoestrogenic state by targeting various points in the hypothalamus–pituitary–ovary axis (Alonso et al., 2024). This can benefit endometriosis lesions directly by reducing proliferation and increasing apoptosis (Gomes et al., 2009; Kiba et al., 2015). However, the effects of this hypoestrogenic state extend beyond the endometriotic lesion itself, decreasing endometrial innervation (Tokushige et al., 2008), and overall menstrual volume (Niu et al., 2021). Whilst these effects can be beneficial for the management of endometriosis-associated symptoms, the lack of specificity of hormonal agents can also affect overall tolerability and suitability for long-term use. This is particularly relevant for second-line agents such as gonadotropin-releasing hormone analogues, which can result in vasomotor symptoms and a loss of bone mineral density (Della Corte et al., 2020). In an attempt to increase specificity, next-generation hormonal agents currently under development are focussing on the known difference in oestrogen metabolism between eutopic and ectopic tissues (Mercorio et al., 2022). By inhibiting specific enzymatic catalysts of in situ oestrogen biosynthesis in the endometriosis lesion itself, systemic effects of hormonal treatments may be ameliorated. Whether this will result in a clinically meaningful outcome remains to be seen, with results of a phase II clinical trial inhibiting steroid sulfatases (NCT01631981) never published. Similarly, while reporting positive data from phase I clinical trials assessing the safety and tolerability hydroxysteroid dehydrogenase type 1 (NCT03709420), efficacy is yet to be established and will be assessed in ongoing phase II trials (NCT05560646). Whether the specificity of next-generation hormonal agents would be sufficient to prevent a contraceptive action remains speculative, but could represent great utility for managing endometriosis in those attempting to conceive (Barra et al., 2019).

The paucity of non-hormonal therapeutics available to treat endometriosis is not reflective of a lack of interest or volume of preclinical research, but instead the limited translational success linked to poor quality and non-representative models of disease often relying on endometrium (Groothuis, 2022). Clinical translation of therapies, particularly in the context of non-malignant disease, relies on the minimisation of adverse effects. There has been increasing interest in precision medicine in endometriosis by repurposing targeted agents developed for cancer (Hung et al., 2021) due to the identification of cancer-associated mutations in endometriosis tissues (Anglesio et al., 2017). However, similar mutations have been found in eutopic endometrium, limiting their specificity (Suda et al., 2018). For instance, despite growing data suggesting that PI3K/AKT and MAPK represent targets of priority for endometriosis treatment (Bao et al., 2022), inhibitors of these pathways have been shown to be more cytotoxic to cells from eutopic endometrium than endometriosis lesions (Lavogina et al., 2019). Future attempts to identify precision targets for endometriosis treatment should consider the differences between eutopic and ectopic tissues and utilise the biological drivers specific to endometriotic lesions instead of endometrial tissues, as has been observed in the literature (Nayak et al., 2022). Furthermore, preclinical testing of these agents should be conducted on endometriotic models as opposed to endometrium (Churchill et al., 2023), including complex ex vivo models (such as organoids or organ-on-a-chip derived from endometriotic tissues, as reviewed in Gołąbek-Grenda and Olejnik, 2022), or in vivo (as reviewed in Zeng et al., 2024).

### The underappreciated value of non-endometrium endometriosis samples

There is increasing evidence that parenchyma and the surrounding local microenvironment is not merely a passive bystander, but an active contributor to endometriosis progression. Mesothelial cells are known to be present within endometriosis lesions (Kerner et al., 1981) and undergo mesothelial-to-mesenchymal transition to promote disease progression and fibrosis (Yan et al., 2020). Even in areas without lesions of endometriosis, retroperitoneal adipose tissue from people with endometriosis demonstrate higher amounts of fibrosis, angiogenesis, and immune infiltration than controls, likely mediated by the systemic proinflammatory milieu initiated by disease (Kubo et al., 2021). Microenvironmental cues themselves seem to promote disease progression, with mere surgical translocation of murine endometrium into the peritoneum sufficient to induce an upregulation of immune, fibrosis, and angiogenesis pathways akin to endometriosis lesions (Li et al., 2024). Microenvironment-relevant control biospecimens separate from the endometrium are an under-represented and underappreciated source of valuable biological insight in endometriosis.

### Diagnosis and prognosis

Peripheral blood contains great diagnostic potential for endometriosis. Circulating cell-free non-coding RNAs, such as miRNAs and long-non-coding RNAs (lncRNAs), are abundant in blood and play important roles in normal inter-cell communication (Ramón Y Cajal et al., 2019). Both miRNAs and lncRNAs are dysregulated in endometriosis patients versus controls and have been implicated in disease pathogenesis via their involvement in inflammatory, immune, and hormonal responses (Hon et al., 2023; Ghafouri-Fard et al., 2020; Abbaszadeh et al., 2023). The difference in non-coding RNAs present in peripheral blood of those with and without endometriosis is of great interest in the identification of specific biomarkers for non-invasive diagnosis (Vanhie et al., 2024; Shan et al., 2022). Recent characterisation of the circulating miRNA transcriptome using plasma samples from 153 patients with endometriosis has informed a diagnostic signature of 86 miRNAs in adults of undisclosed phenotype (Bendifallah et al., 2022). Beyond diagnosis, circulating non-coding RNAs may also hold prognostic value. The downregulation of lncRNA, LINC01456, in serum is associated with advanced disease stage and is predictive of endometrioma recurrence (Song et al., 2024). Serum miR-1307-3p levels have also shown clinical value, predicting the efficacy of perioperative dienogest treatment for ovarian reserve preservation after endometrioma cystectomy (Yabuki et al., 2024).

Similar attempts to utilise blood cell-free DNA (cfDNA) for diagnosis have yielded conflicting results (Yuwono et al., 2022; Alonso et al., 2022; Haouzi et al., 2018). Phenotype-specific differences in cfDNA concentration and fragmentation have been observed (Yuwono et al., 2022; Haouzi et al., 2018), supporting the need for large, unbiased sample cohorts to account for endometriosis heterogeneity. While those with endometriosis showed significantly decreased cfDNA fragmentation compared to controls (Yuwono et al., 2022), attempts to increase test specificity by investigating cfDNA methylation signatures relied upon endometrial methylation patterns, which have not been investigated in endometriosis tissues (Yuwono et al., 2022; Alonso et al., 2022).

Peripheral blood-based biomarkers offer unique benefits for diagnosis compared to endometrial biospecimens like lavage or menstrual effluent. Blood tests already form a cornerstone of medical diagnostics and would require minimal additional infrastructure to implement, as may be necessary with patient-collected effluent samples. As a circulating liquid biopsy, blood may provide a more comprehensive window into the systemic effects of endometriosis, or more accurately detect lesions beyond the pelvis, such as thoracic endometriosis (Kiss et al., 2020). To date, attempts to establish an endometriosis diagnostic signature in blood have been more accurate than in menstrual effluent, with higher overall sensitivities and specificities (96.8 vs 87.5 and 100.0 vs 91.7, respectively) (Nayyar et al., 2020; Bendifallah et al., 2022). In addition, analysis of peripheral blood is specifically advantageous compared to menstrual samples, particularly for patients who do not menstruate due to hormone-induced amenorrhea (Kim et al., 2022; Prosperi Porta et al., 2021; Ferrando et al., 2021), obstructive anatomical variations (Vercellini et al., 2024b), menopause (Haas et al., 2012), and those post-hysterectomy (Soliman et al., 2017).

### Disease aetiology

The assumption that evidence of an endometrial origin of endometriosis lesions for some patients precludes any other explanation of pathogenesis is misguided. There is equivalent evidence of a non-endometrial origin of endometriosis, as shown by case reports of endometriosis in cisgender men and uterine agenesis. The true incidence of both these entities remains elusive due largely to a lack of consistency in research methods and reporting. For example, despite the 47 case reports of endometriosis arising in people with MRKH syndrome (Wang et al., 2017; Steinmacher et al., 2022; Marsh et al., 2013; Tian et al., 2022; Mok-Lin et al., 2010; Cho et al., 2009), the majority of patients (45/47, 95.7%) retained uterine remnants, of which all cases which underwent histological review contained functional endometrium (35/35, 100%) (Steinmacher et al., 2022; Marsh et al., 2013; Tian et al., 2022). In fact, only two isolated cases of endometriosis have been reported in the context of complete uterine agenesis: one endometrioma (Cho et al., 2009) and one peritoneal endometriosis (Mok-Lin et al., 2010), the latter of which was not histologically confirmed due to lesion ablation. Similarly, despite 24 case reports of endometriosis in cisgender men and one case in a transgender woman (Coleman-Belin et al., 2024), at least four of these patients presented with innate variations of sex (intersex) characteristics (Nerune et al., 2016; Vaughn and Gonzalez-angulo, 1961; Patel and Doody, 2008; Ohan et al., 2024). In addition, there is one reported case study of peritoneal endometriosis in a prepubescent child with a 46,XY karyotype, female external genitalia, and gonadal dysgenesis. While endometriosis was present histologically, the unicornuate uterine horn was not removed or sampled for functional endometrium (Harris et al., 2024). All these case reports presented with disease in areas of coelomic epithelium, derived from areas of the embryonic Müllerian ducts (Rei et al., 2018). The majority of cases arose with a background of high systemic oestradiol, namely from obesity (4/21, 19.0%) (Rei et al., 2018; Al-Obaidy and Idrees, 2019; Zámečník and Hoštáková, 2013; Balgobind et al., 2019), cirrhosis (2/21, 9.52%) (Jabr and Mani, 2014; González et al., 2014), gender-affirming care (1/21, 4.76%) (Coleman-Belin et al., 2024), or cancer-related androgen deprivation therapy (10/21, 47.6%) (Fukunaga, 2012; Taguchi et al., 2012; Pinkert et al., 1979; Beckman et al., 1985; Martin and Hauck, 1985; Oliker and Harris, 1971; Schrodt et al., 1980; Young and Scully, 1986; Scully, 1981). Additionally, one case of an endometriotic cyst showed areas of direct transformation from native mesothelium to endometriotic epithelial cells (Zámečník and Hoštáková, 2013).

The concept that endometriosis may arise from a metaplastic process of mesothelial cells has previously been proposed in the literature (Zheng et al., 2005; Konrad et al., 2019). A histological review of 110 endometriomas revealed 34 cases with areas of direct transition from normal native epithelium to endometriosis (Zheng et al., 2005), an observation that is yet to be recorded in either superficial or deep infiltrating peritoneal lesions. Interestingly, all observed cases of endometriosis in non-primate (and therefore non-menstruating) animals have also presented as endometriomas (5/5 dogs, 1/1 guinea pigs), which may further support that endometriomas are more likely to develop independently of endometrium than other phenotypes of endometriosis (Demirel, 2017; Paiva et al., 2015; Baldi et al., 2017; Bartel et al., 2011). However, evidence of clonal metastasis from endometrioma to peritoneal locations indicates that the development of diverse phenotypes could arise from a single initiating event, which cannot, at this stage, exclude metaplasia (Praetorius et al., 2022). Therefore, while to date there is an absence of evidence that metaplasia may initiate peritoneal endometriosis, this does not denote evidence of absence.

### Treatment

Investigating parenchymal tissues adjacent to endometriosis lesions may provide novel insights into disease mechanisms and development. For example, in cancer, transcriptional profiles of ‘normal’ tumour-adjacent tissue have been found to have prognostic value (Kim et al., 2023; Oh and Lee, 2023), and there are increasing efforts to target native microenvironment fibrosis, neovascularisation, and inflammation to improve patient outcomes (Xiao and Yu, 2021).

Similar findings are emerging in other inflammatory diseases. In Crohn’s disease, adjacent mesenteric adipose tissue actively contributes to disease development and fibrosis (Huang et al., 2022), and may have a direct effect on the efficacy of infliximab treatment (Shen et al., 2018). Clinical trials targeting resident synovial fibroblasts are currently underway in rheumatoid arthritis (Siebert et al., 2020). However, this notion is almost entirely unexplored in endometriosis. Future endeavours to classify and potentially target adjacent tissues in endometriosis lesions may hold therapeutic benefit for patients and should be a priority for prospective research.

### Conclusion

With evidence supporting both endometrial-dependent (Suda et al., 2018) and endometrial-independent pathogenesis (Zheng et al., 2005), neither theory provides sufficient evidence to preclude the other, and they likely represent separate aetiological avenues of distinct subtypes of endometriosis. By focusing predominantly on endometrial-dependent pathogenesis, current preclinical research is ensuring that the collective understanding of endometriosis aetiology remains incomplete. Furthermore, the paucity of studies containing parenchymal tissues as a biological control demonstrates an underestimation in the literature at large of the contribution of the local microenvironment to disease development and progression.

**Figure 3.**
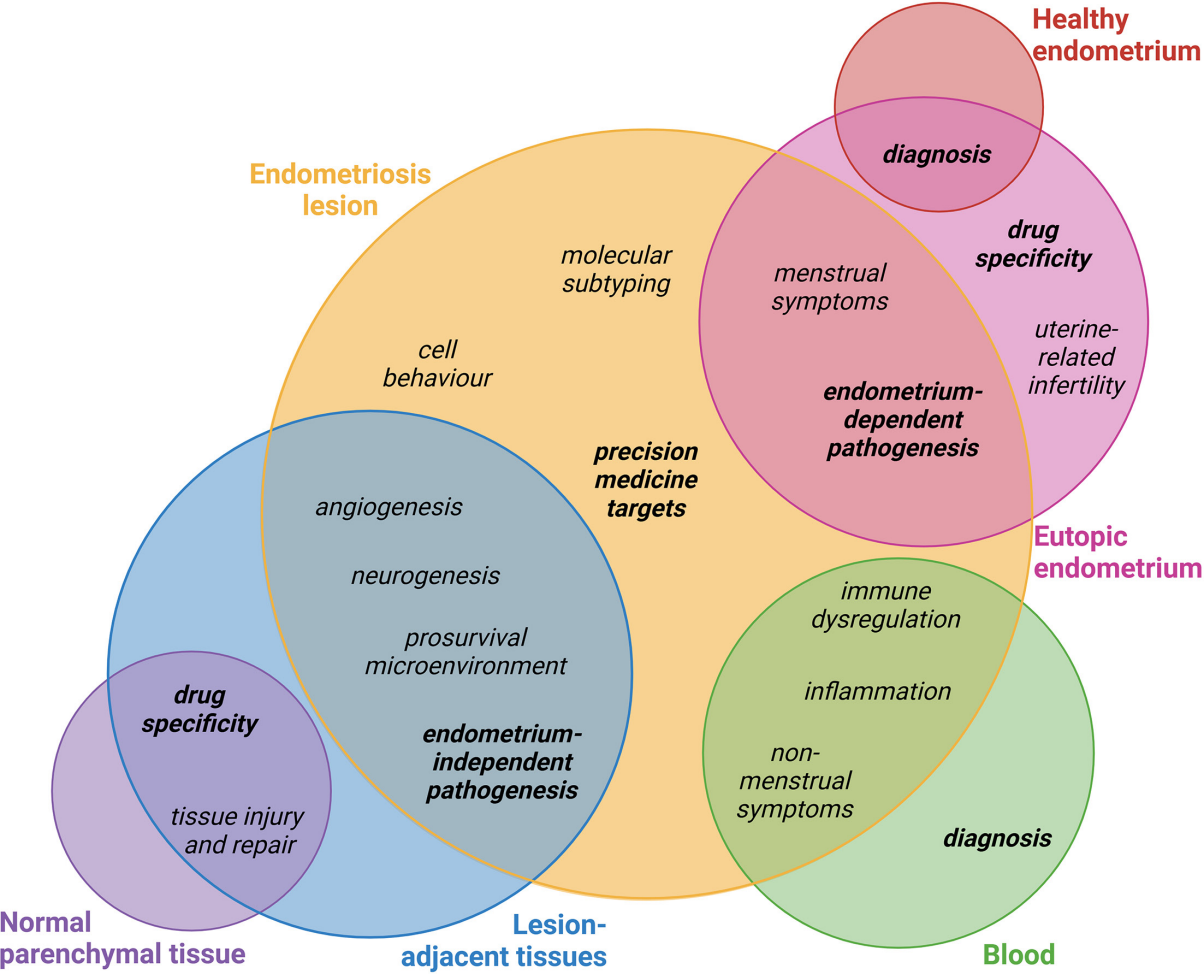
Proposed biospecimen types which could be used to answer key research priorities in endometriosis. Circles corresponding to biospecimen type are not scaled. Topics in bold discussed in this review. This figure was created using BioRender.com.

It is clear that key research questions can be answered using both endometrial and non-endometrial biospecimens, and that incorporating diverse perspectives and methodologies in approaching these questions remains invaluable. Figure 3 represents some of these questions and recommendations of how both endometrial and non-endometrial biospecimens may be utilised for research in these fields.

The current analysis likely does not account for every dataset generated for endometriosis research and cannot report on data generated but not publicly shared. However, by analysing the publicly available data from two of the most popular sites for data sharing, this review found a sizeable overlap of available data on both sites (75/245, 30.20%), indicating that there was a moderate saturation and that the main findings of this analysis represent a true bias in field towards endometrium-derived data in endometriosis research. Despite an increasing understanding in recent years of the innate biological differences between endometriosis and endometrium, the number of biospecimens relying solely on endometrium published each year consistently remains approximately 50% (Figure 2C). The lack of temporal shift towards more inclusive sampling is indicative of a persistent bias in the field of both thought and methodology. Future research should prioritise collecting broader types of specimens, including but not limited to endometriotic lesions, adjacent parenchymal tissues, patient and menstrual phase-matched eutopic endometrium, peripheral blood, peritoneal fluid, follicular fluid, and vaginal swabs. These biospecimens should also include as much matched clinical data as possible such as surgical phenotype, menstrual phase, and use of hormonal treatments. Broader sampling will provide a more comprehensive view of the various biological processes at play within endometriosis as a complex and multisystem disease.

In conclusion, endometriosis does not represent ‘ectopic endometrium’, and the phrase should be abandoned. While there is some value in the presence of eutopic endometrium in endometriosis research, the scale of representation is inordinate. The rise in data-sharing and secondary analysis increases overall accessibility of research by conserving time, money, and resources, but there is a responsibility to collect and accurately report the source of more diverse biospecimens than is currently accounted for in the field of endometriosis. Likewise, researchers utilising existing datasets should carefully review the origin of samples and select a dataset which captures the type of biospecimen necessary to answer the research question at hand, including relevant controls. Unravelling the complexities of endometriosis pathophysiology and identification of novel treatment options will rely on unbiased experimental design and evolution of thought beyond comparing eutopic endometrium to endometriotic lesions, leading to better management and outcomes for all those affected by endometriosis.

## Additional files

Supplementary file 1. Review of publicly available datasets for endometriosis. Contains search methods, exclusion criteria, and links to the source and publications relating to all publicly available datasets as described in the article. Year of publication, platform, biospecimen type and use of relevant controls also listed.
